# Study of the Ecological Footprint and Carbon Footprint in a Reverse Osmosis Sea Water Desalination Plant

**DOI:** 10.3390/membranes11060377

**Published:** 2021-05-21

**Authors:** Federico Leon, Alejandro Ramos-Martin, Sebastian Ovidio Perez-Baez

**Affiliations:** Process Engineering Department, Universidad de las Palmas de Gran Canaria, 35001 Las Palmas de Gran Canaria, Spain; alejandro.ramos@ulpgc.es (A.R.-M.); sebastianovidio.perez@ulpgc.es (S.O.P.-B.)

**Keywords:** ecological footprint, carbon footprint, reverse osmosis, desalination

## Abstract

The water situation in the Canary Islands has been a historical problem that has been sought to be solved in various ways. After years of work, efforts have focused on desalination of seawater to provide safe water mainly to citizens, agriculture, and tourism. Due to the high demand in the Islands, the Canary Islands was a pioneering place in the world in desalination issues, allowing the improvement of the techniques and materials used. There are a wide variety of technologies for desalination water, but nowadays the most used is reverse osmosis. Desalination has a negative part, the energy costs of producing desalinated water are high. To this we add the peculiarities of the electricity generation system in the Canary Islands, which generates more emissions per unit of energy produced compared to the peninsular generation system. In this study we have selected a desalination plant located on the island of Tenerife, specifically in the municipality of Granadilla de Abona, and once its technical characteristics have been known, the ecological footprint has been calculated. To do this we have had to perform some calculations such as the capacity to fix carbon dioxide per hectare in the Canary Islands, as well as the total calculation of the emissions produced in the generation of energy to feed the desalination plant.

## 1. Introduction

Canary Islands water demand has been a historical problem solved by desalination. Due to this Canary Islands was a pioneering place in the world in desalination issues, allowing the improvement of the techniques and materials used.

Desalination has a negative aspect, in that the energy costs of producing desalinated water are high. Moreover, it is necessary to add the peculiarities of the electricity generation system in the Canary Islands, which generates more emissions per unit of energy produced compared to the continental generation system.

In this study, our objective is to select a desalination plant to study this desalination procedure and energy consumption. It is located on the island of Tenerife, specifically in the municipality of Granadilla de Abona, one seawater reverse osmosis plant very well known by us including their technical characteristics to take it as example and to study the ecological footprint.

To do this we have had to perform some calculations such as the capacity to fix carbon dioxide per hectare in the Canary Islands, as well as the total calculation of the emissions produced in the generation of energy to feed the desalination plant.

### Literature Review

The ecological footprint (EF) is a measure of the area of biologically productive land and water that is required for an individual or an activity to produce all the resources it consumes and to absorb the waste it generates, using prevailing technology and resource management practices [1].

The EF is defined as the total ecologically productive area necessary to produce the resources consumed by an average citizen of a given community, as well as the one necessary to absorb the waste that this generates, without regard to the location of these areas. The ecological footprint corresponds to the area of ecologically productive territory (crops, pastures, forests, or aquatic ecosystem) necessary to produce the resources used and to assimilate the waste produced by a defined population with a specific standard of living indefinitely, wherever this area is located [2].

It will therefore be a variable directly proportional to resource consumption and population, as well as to waste generation, so we could say that the ecological footprint is responsible for determining which productive soil area is necessary to maintain a specific population indefinitely, wherever the soil is located, highlighting the territorial character with respect to the population [3].

The latest results from the National Footprints Accounts (NFA) 2018 Edition for the year 2014 indicate that humanity’s EF is 1.7 Earths, and that global ecological overshoot continues to grow. While improved management practices and increased agricultural yields have assisted in a steady increase of Earth’s biocapacity since 1961, humanity’s EF continues to increase at a faster pace than global biocapacity, particularly in Asia, where the total and per capita EF are increasing faster than all other regions [4].

Situations in which total demand for ecological goods and services exceed the available supply for a given location, are called ‘overshoot’. ‘Global overshoot’ indicates that stocks of ecological capital are depleting and/or that waste is accumulating. As the methodology keeps being improved, each new edition of the NFA supports the findings of a global overshoot [5].

Global Footprint Network’s National Footprint Accounts, supported and used by more than 70 major organizations worldwide, contain the most widely used national accounting methodology today. The National Footprint Accounts calculations are undergoing continuous improvement as better data becomes available and new methodologies are developed [6].

The ecological footprint computes the yields per unit of surface of the primary product flows to find the area necessary to be able to carry out a certain activity. Biological capacity or biocapacity is the supply created by the biosphere, this implies the measure of the amount of biologically productive land and the maritime zone available to provide the services of the ecosystems that humans consume [7].

A reduction in energy consumption will have a direct impact on environmental improvement and we study this through the carbon footprint produced by these desalination plants and their ecological footprint, the latter as a future line of action [8,9].

Energy consumption and urban population reveal moderate increasing returns to scale, while GDP per capita exhibits decreasing positive returns [10,11].

Canary Islands and island areas in general are very complicated about ecological footprint due to the traits that are geographical isolation and scarce resources. Therefore emphasizing the sustainability of the Canary Islands’ ecological systems is a key step for achieving sustainable development goals [12].

It is important to study the challenges of calculating the ecological footprint of Canary Islands, because they are areas smaller than the continental territory. Ecological footprint analysis provides a useful tool for measuring the environmental load of urban restructuring [13].

Both footprint and biocapacity are estimated for different uses of the territory and can account for the variation in estimated average territorial productivity for different purposes, therefore they are expressed in terms of average global hectares, territory or biologically productive maritime zone, this unit of measure being the global hectare (Gha). A global hectare will have productivity equal to the average productivity of the 11.2 million bio productive hectares on the planet. Productivity does not refer to the rate of biomass production, such as net primary production (PNP). In this case, productivity is the potential to achieve maximum agricultural production with a certain level of inputs. Thus, a hectare of highly productive land is equal to more global hectares than a less productive hectare of land. Global hectares are standardized so that the number of hectares of land and sea on this planet is equal to the defined global number of hectares [3]. Thanks to this concept of global hectares, it is possible to compare the ecological footprint and biocapacity of different countries of the globe with different farmland, grazing lands and forests. The conversion factors applied to achieve this purpose are equivalence factors (constant for all countries and for a given year) and performance factors (country-specific and year-specific); translate each of the hectares of biologically productive areas into global hectares.

Equivalence factors represent the global average productivity potential of a given bio productive are considering the concept of global average productivity of total bio productive zones. The equivalence factors of farmland, forests, grasslands, and infrastructure-occupied areas are derived from the global indices established by Global Agri-Ecological Zones (GAEZ) 2000, a spatial model of the potential of agricultural yields (Table 1).

This factor describes the possible achievable yield by crops in an area with a theoretical assumption of inputs such as water and fertilizers, regardless of management practices or types of biomass production.

Performance factors in each country seek to quantify the relationship between a biologically productive zone and the global average of the same bio productive zone. In other words, it implies the relationship between the production of goods in a given category of a country, nation, or territory, with their national yields and the amount of territory that would have to be established to produce the same goods with average global yields; therefore, each country has its own set of performance factors, which must be recalculated each year [8,9].

The performance factor reflects the prevailing technology and management practices in the territory, as well as the productivity of domestic renewable resources. If agricultural production per hectare in a country depends on soil fertility as well as farming methods, the yield factor will reflect the national average with subsequent weighting of existing climatic zones. Given that there are many countries that have different and marked climate zones, it is necessary to reflect with greater resolution the accuracy of their actual productivity in regional or local analyses; recalculating, as far as possible, the performance factors to reflect the circumstances of each place. From an operational point of view, the procedure for calculating the ecological footprint in the strict sense is based on three stages.

In the first stage the average annual consumption per person of the specific goods to be considered is estimated, it is classified consumer goods into five main categories: food, housing, consumer goods, transport, and services. Having access to regional or national data and dividing consumption by the number of inhabitants.

Second, the appropriate area per capita (aa) is estimated to produce each type of good. This is done by dividing the average annual consumption of each good (in kg/per capita) by the average annual productivity per Gha ($p_{i}$ en kg/Gha):

(1) $${\mathrm{aa}}_{i}=c_{i}/p_{i}$$

After this, we obtain the total ecological footprint per capita by adding up all the biologically productive areas for the *n* goods and services:

(2) $$\mathrm{HE}\;=Ʃ{\;\mathrm{aa}}_{i}$$

Finally, we obtain the total ecological footprint of a particular population, this being the product of the previous expression by the total number of inhabitants N:

(3) $${\mathrm{HE}}_{T}=N\times \mathrm{HE}$$

This way one can calculate the ecological footprint [6,7,8].

First, the main advantage of ecological footprint as an indicator is in the simple unit of measurement of this, the hectare, which allows us to make comparisons between different footprints in different places in the world. Thus, we can compare the cost of producing one kilogram of tomatoes with the cost of consuming one liter of kerosene. Therefore, the ecological footprint gives that easy comparison between products or services no less antagonistic. We can list many advantages:

- It defines sustainability objectives in specific and measurable terms by providing great information to Nongovernmental Organizations (NGOs) and governments to establish sustainability-oriented measures, creating a meaningful context for decision-making.
- See the areas where there is the greatest room for improvement in reducing consumption and promoting sustainability. Once the mostly guilty camps have been analyzed, the ecological footprint also allows us to examine the costs of reducing it.
- It develops activities that maintain the citizen’s interest in ensuring a sustainable future. It is a concept that is easily conveyed and helps to raise awareness of the importance of sustainability and promote initiatives or actions for it. This footprint provides more information to existing sustainability projects.
- Create strategies to speed up the process. It promotes the reduction of the ecological footprint by improving the quality of life of people. Create a platform to be able to properly plan important topics such as the field of housing, transport or energy and its infrastructures.

As for the disadvantages, the calculation of the ecological footprint is complex and limits its handling. As for the statistical methods used, they have been quite criticized because they require acceptance of many hypotheses, many questionable.

It is also an indicator designed for use at the national level, as it is the way in which reliable data is obtained and handled for the calculation being more difficult to ensure the reliability of these at the regional level. The data in question are imports and exports, food consumption, global energy consumption, etc.

This indicator rewards the replacement of original ecosystems with high productivity agricultural monocultures so that biocapacity per hectare increases. In this way it implies that organic agriculture, which has lower productivity, produces a higher ecological footprint than intensive crops.

On the other hand, it should be noted that no other impacts are counted such as water pollution, erosion, soil pollution, air pollution (except CO_2_).

Finally, this indicator assumes that soil productivity on farms and livestock does not decrease over time; obviously if it decreases, using these itself and by erosion and pollution [8,9,10].

## 2. Materials and Methods

In this section it is described the methods of calculation of the ecological footprint in the Granadilla de Abona desalination plant. The production of desalinated water, with all the daily tasks that come with its production generate an impact on its environment, from the capture of seawater, the consumption of electricity, the generation of waste, the use of a surface of land for this purpose, the generation of gases such as CO_2_ in the generation of electricity consumed, etc.

In the Canaries it is necessary the desalination of large amounts of water to allow the development of the islands considering that almost all the electricity produced is from fossil fuels, it is about converting oil into water on a planet that demands to reduce emissions. So, we can see that it is about solving local CO_2_ problem, water scarcity, helping to worsen a global problem such as air pollution. The Canary Islands’ dependence on fossil fuels is extremely high due to its status as islands and because it does not have facilities such as gas storage or nuclear power plants, to give some examples. Altogether, water desalination in the Canary Islands means more than 770,000 MWh, equivalent to more than 180,000 tons of fossil fuels each year (3600 barrels per day), at a cost of more than 100 million euros each year and the emission of more than 450,000 tons CO_2_ into the atmosphere each year [11]. Only 5.6% of energy production in the Canary Islands is produced by renewable energy sources. As the Canary Islands, a region with resources such as wind or sun, it is vitally important to study how to apply these energy sources in the desalination process to reduce energy demand from other non-renewable sources [14,15,16].

The impact of brine discharge and greenhouse gas emissions need to be estimated in a full valuation based on the consequences on the natural environment and on the basis of technology. In the Canary Islands, the preservation of marine habitats is of vital importance due to their economic and social transcendence, since tourism is the main economic source of the islands, as well as the importance of fishing, albeit to a lesser extent, than the previous sector.

Landscape degradation is difficult to reverse, so it is very important to minimize any damage to the environment.

The loss of biocapacity related to brine discharges directly affects reducing biodiversity on the coasts in the face of physical-chemical modification of the marine environment, or indirectly, by reducing catches in fisheries and by decreasing and losing habitats and species.

The methodology to calculate the carbon footprint of the current and future energy mix, considering the sum of the energies of each technology and its emissions mix factor is the following [8]:

HC_MIX_ = ΣE_i_ FM_i_ (4)

The current energy and future energy of the technologies of the energy mix are the following:

E^1^t_MIX_ = ΣE_i_ in the initial moment (5)

E^2^t_MIX_ = ΣE_i_ in the final moment (6)

being HC_MIX_: carbon footprint of the energetic mix (tCO_2_), E^1^t_MIX_: actual energy of the energetic mix technologies (kWh), E^2^t_MIX_: future energy of the energetic mix technologies (kWh), E_i_: energy of each technology (kWh), P_t_: percentage of use of each technology in the energetic mix and FM is the factor mix, calculated for each technology and island with the total consume of energy per island, associated to the carbon footprint and the percentage of one technology in the energetic mix, including renewable and non-renewable energies [8].

### Energy Analysis, Costs, and Emissions of Desalinated Water in the Canary Islands

The cost of desalination is quite high when compared to freshwater exploitation where energy is only consumed when pumping water from where the consumer is extracted. In the Canary Islands there is a predominance of the use of reverse osmosis, this process being 77% of the total desalinated water [13].

In the process of desalination of water by reverse osmosis energy is consumed in the pumping system, the operation before treatment and to generate pressure in the reverse osmosis stage, this being the most important process. High energy costs include the consequent environmental impact on energy generation, where greenhouse gases are emitted.

At the end of 1970, the first reverse osmosis desalination plants consumed up to 20 kWh/m^3^. Over the years this process was perfected, getting better materials, more efficient membranes, the use of energy recovery devices, etc. It managed to reduce energy consumption to about 3.5 kWh/m^3^ in the late 1990s. Today the energy consumption is technically possible to place it below 2 kWh/m^3^.

In short, energy is the highest cost component in the operation of a reverse osmosis desalination plant and at the same time is the factor with the greatest potential for cost reduction. The amount of energy in the overall computation of a reverse osmosis plant varies depending on its operating and location parameters. In our case we will make an average estimate of values to obtain the consumption of our Granadilla desalination plant.

Below is a graph of the typical distribution of total energy cost in reverse osmosis desalination plants. It is noted that the largest contribution is directly related to the high-pressure pumping required by this technology, with 84.4%.

The production costs of one cubic meter of desalinated water are conditioned by several factors: facility depreciation, maintenance, chemicals, personnel, taxes, membrane change and energy; the latter is the most decisive part of the overall analysis of investment in the production of desalinated water in the Canary Islands assuming 41% of the total costs. Next, we will look at a graph of the factors and their percentage in the final cost of desalinated water [11].

As mentioned above the model of electric production in the Canary Islands is almost entirely produced from fossil fuels. In the case of the conventional generator park, a yield of between 32% and 36% is estimated; emissions related to an average thermal power plant are estimated by the public administration in 0.402 kg CO_2_/kWh [14].

When calculating the actual energy consumption of desalinations in the Canary Islands we are faced with a complicated task due to the lack of information and we will have to approximate some values to have a good perception of reality and allow us to propose alternative scenarios to reduce the ecological footprint.

It should be noted that desalinated water is produced on the coast, so it will then have to be pumped and stored for later distribution so that the energy bill increases by 1 or 2 kWh more for every 1000 L, as well as water losses and subsequent water purification.

According to data provided by the Directorate-General for Industry (DGIE) it is obtained that the average specific energy consumption is at 4.89 kWh/m^3^ [6].

Below will be a table with the energy consumption of each island and the percentage of this energy used for desalination. It is necessary to consider that it is based on data present in the Energy Statistics of the Canary Islands along with information of the hydrological plans of each island. It should also be clarified that we start from an assumption in which all desalinated water is performed by reverse osmosis, when a large part is, but a small amount is desalination or other techniques. Finally, let us determine an average consumption of 4.89 kWh/m^3^ (Table 2).

Next, let us study the emission coefficients that are assigned to the power generation on each of the different islands. Firstly, we have observed the emissions data provided by Endesa Generation, which is the company that has a monopoly on power generation in the Islands from power plants that use fuel for it. The fact is 0.31 kg CO_2_/kWh [17].

Subsequently we have consulted the emission values on the Spanish Electric Network website and obtained values for each of the islands separately. To obtain an average emissions data from power generation, we will select all daily emission coefficients per island, taking a value every two hours, and obtaining the daily average of a random working day to be able to further approximate this emission coefficient. Specifically, we will select 6 October 2020. We will tabulate these values in Table 3 to get the exact value in each case.

Based on what is obtained in Table 3, we can see how the coefficient of emissions by the generation of energy in the Canary Islands is between values of 0.5 and 0.7 t/MWh, highlights that the island of Tenerife has the lowest coefficient, enhanced by the presence of more efficient and less polluting energy generation systems along with the presence of CO_2_ renewables. On the opposite hand in Lanzarote and La Gomera we have coefficients of approximately 0.7 t/MWhCO_2_, partly due to polluting generation systems and a low presence of renewable energy. It should be noted that these measurements have been made for a specific day and no aspects related to meteorology have been thoroughly analyzed that can condition the amount of energy generated cleanly through renewables, but if it is a value very close to the actual situation of the islands throughout the year. By taking this data on the same day for each island we ensure that there is equality or parity in the climate aspect in the archipelago which makes energy generation through renewables equitable. Finally, it is worth highlighting the value of 0.525 t/MWh, which corresponds to the emission factor of the island of Tenerife and is the one that we will use as a reference when CO_2_ performing our calculations later [18].

Below is a table of emissions related to desalination on each of the islands considering the coefficient related to energy generation in each of the different islands calculated above (Table 4).

According to the estimates made with the data provided by the hydrological plans of the Canary Islands and the Energy Statistics of the Canary Islands it is obtained that the annual production of desalinated water in the Canary Islands is 216.41 ${\mathrm{hm}}^{3}$, for which it is necessary to use 1,058,240 MWh. Generating this volume of desalinated water generates a total of 694,445 tons of CO_2_.

About the calculation method of the ecological footprint of the Granadilla desalination station in Abona, the impact associated with natural resource consumption and waste production shall be determined based on emissions from CO_2_ each consumption or type of waste produced by the desalination plant. These emissions into the environment will then be translated into canary forest area necessary to assimilate them (Table 5).

Emission factors obtained from various sources are used for the calculation of the CO_2_ emissions, some of which have been discussed above. To calculate directly from consumption, in some cases emissions are obtained by multiplying consumption by emission factors. This usually applies to water consumption, consumption associated with building construction, electricity consumption, heat energy or natural gas consumption, among others.

In our case we will calculate the area, in hectares (ha) of forest or forest area required to absorb the one produced by the consumption of resources and the production of waste mentioned above. From the amount of emitted into the atmosphere, dividing by the fixing capacity of the Canarian forest mass, the required forest area is obtained. To this amount of forest will also be directly added the space occupied by the desalination plant.

With the data obtained by the Government of the Canary Islands in its last measurement of greenhouse gases in 2008 in the Canary Islands, we can see that the emissions of 13,517,320 tons. It is also said that carbon fixation by forest masses is around 16.13% of the CO_2_ emissions.

We get that a total of 2,180,343 tons are fixed in the year of the study. To compare this measure, we must obtain the capacity to fix the forest masses by hectares, so we will have to divide the emissions of CO_2_ per the area of the Canary Islands into hectares, which are about 749,300 Gha [19]. So, finally it is obtained 2.91 $\frac{{\mathrm{tons}\;\mathrm{CO}}_{2}}{\frac{\mathrm{ha}}{\mathrm{year}}}$.

Considering the above explanations, the ecological footprint is calculated using the following formula:

(7) $$\mathrm{Footprint}\left(\frac{\mathrm{ha}}{\mathrm{year}}\right)=\frac{emissions\;\left({t\;\mathrm{CO}}_{2}\right)\;\;}{C.\;\;fixing\;(\frac{{t\;\mathrm{CO}}_{2}}{\frac{\mathrm{ha}}{\mathrm{year}}})}+\frac{emissions\;edif.\;\left({t\;\mathrm{CO}}_{2}\right)}{C.\;\;fixing\;(\frac{{t\;\mathrm{CO}}_{2}}{\frac{\mathrm{ha}}{\mathrm{year}}})}$$

To compare different ecological footprints, the global hectare (Gha) is used which is defined as the average global capacity to produce resources and absorb waste. To express the results to this extent, the different types of existing areas must be normalized.

Equivalence factors translate a specific type of land into the universal unit for the productive area (Gha) and are based on measures of land productivity based on their uses and years.

Next, we will proceed to the calculation of emissions related to electricity consumption in a very simple way, based on the consumption data mentioned above, the daily production of the desalination plant in $m^{3}\;$ and finally the appropriate emission factor. The following formula makes clear the calculation to be made:

Emissions (kg CO_2_) = consumption (kWh/year):

(8) $$\mathrm{emission}\;\mathrm{factor}=(\frac{{\mathrm{kgCO}}_{2}}{\mathrm{kWh}/\mathrm{year}})$$

## 3. Results and Discussion

As mentioned above, the average consumption value of desalination plants in the Canary Islands is 4.89 kWh/m^3^. The Granadilla de Abona desalination plant has a production capacity of 14,000 m^3^/d and this is going to be the value we take as a reference. Finally, as mentioned above we will select the coefficient of emissions obtained in Spanish Electric Network for the day 13 October 2020 on the island of Tenerife being this 0.525 kg/kWh or CO_2_ also 0.525 tCO_2_/MWh.

(9) $${\mathrm{Emissions}}_{\left(Electric\;consumption\right)}\left({\mathrm{kgCO}}_{2}\right)=24,987,900\left(\mathrm{kWh}/\mathrm{year}\right)\times 0.525(\frac{{\mathrm{kg}\;\mathrm{CO}}_{2}}{\mathrm{kWh}})$$

(10) $${\mathrm{Emissions}}_{\left(Electric\;consumption\right)}\left({\mathrm{kgCO}}_{2}\right)=13,118,648{\;\mathrm{kg}\;\mathrm{CO}}_{2}/\mathrm{year}$$

Finally, the Granadilla de Abona desalination plant emits 13,119 tons of CO_2_ to the atmosphere due the energy consumed.

Secondly, let us calculate the emissions related to the construction of the building or that of the desalination plant in this case. According to the MIES report of the Polytechnic University of Catalonia the emission factor used for the construction of buildings is 520 kg CO_2_/m^2^ of buildings, considering that the useful life for constructions of typology similar to those existing in the Granadilla de Abona desalination plant is estimated between 15 and 50 years, as set out in Royal Decree 1247/2008, of 18 July, which approves the instruction of structural concrete [20,21,22,23,24,25,26,27,28,29,30,31].

Let us choose a mid-life term by selecting 35 years for our calculations so we would have to divide our emission factor by the number of years of service life and thus get the annual factor. It is shown an emission factor of 14.86 kg CO_2_/m^2^ built. Finally, it is calculated the area of the constructions of the Granadilla de Abona desalination plant considering the data collected, such as that of the process surface is 1144 m^2^. It is also necessary to add the surface of the semi-buried deposit of 1695 m^2^ obtained Grafcan database. So, we finally get a total of 2839 m^2^ built:

(11) $${\mathrm{Emissions}}_{\left(Building\right)}\left({\mathrm{kgCO}}_{2}\right)=\mathrm{Area}\;\left(m^{2}\right)\times \mathrm{emission}\;\mathrm{factor}\;(\frac{{\mathrm{kgCO}}_{2}}{m^{2}\times \;\mathrm{year}})$$

(12) $${\mathrm{Emissions}}_{\left(Building\right)}\left({\mathrm{kgCO}}_{2}\right)=2839\left(m^{2}\right)\times 14.86\left(\frac{{\mathrm{kgCO}}_{2}}{m^{2}\times \;\mathrm{year}}\right)=42,187.54\frac{{\mathrm{kgCO}}_{2}}{\mathrm{year}}$$

Finally, it is confirmed that 42.19 tons are CO_2_ emitted for the construction of the different infrastructures of the desalination plant. As for the wastes generated, they will have a less impact on the generation of emissions CO_2_ so we will not consider them in this study.

It is vitally important to know the CO_2_ emissions related to the desalination plant and try to reduce them; this will imply a reduction of the ecological footprint. It is a priority since the Kyoto Protocol, which is a United Nations Protocol on Climate Change and an international agreement aimed at reducing emissions of six greenhouse gases. This document committed industrialized countries to controlling greenhouse gas emissions and setting future targets for progressive reduction of greenhouse gas emissions.

Finally, to obtain the ecological footprint of the desalination plant we will have to add the emissions and divide them by the ability to fix the forest mass in the Canary Islands, which we calculate before calculating emissions, so it would look like this:

(13) $$\mathrm{Footprint}\;\left(\frac{\mathrm{ha}}{\mathrm{year}}\right)=\frac{\;13,119\left({t\;\mathrm{CO}}_{2}\right)\;}{2.91\;(\frac{{t\;\mathrm{CO}}_{2}\;}{\frac{\mathrm{ha}}{\mathrm{year}}})}+\frac{42.19\;\left({t\;\mathrm{CO}}_{2}\right)}{2.91\;(\frac{{t\;\mathrm{CO}}_{2}\;}{\frac{\mathrm{ha}}{\mathrm{year}}})}=4522.75\;\mathrm{Gha}/\mathrm{year}$$

Which translated into global hectares (Gha) would be 6060.5 Gha/year.

The global hectare (Gha) values for each land type will then be displayed based on the calculated ecological footprint (Table 6).

Finally, it is considered the size of the population under study. In the case of the Granadilla de Abona desalination plant it should be noted that the population of this urban center is 70,000 inhabitants. Therefore, it would be an ecological footprint of 0.087 Gha/inhabitant/year.

Comparing with other references it could be confirmed that for an annual production of desalinated water in the Canary Islands of approximately 660,000 m^3^/day and considering an average energy consumption of 3.04 kWh/m^3^, introducing equipment to recover energy from brine, we have a carbon footprint of 1203.84 tCO_2_/day, which means that there are 439,402 tCO_2_ per year and approximately a value of 219,701 Gha/year [8,13].

Considering the surface area of the Canary Islands being 749,300 ha, and population of 2,207,225 habitants, this ecological footprint per person should be a value of 0.1 Gha/person/year and the emissions per inhabitant and year are 0.2 tCO_2_/person/year as [8,13].

Therefore, to reduce the ecological footprint of desalination plants in the Canary Islands, there are several possibilities. Firstly, it could be possible to reduce the cost and emissions in energy generation by burning fuels in thermal power plants in the Canary Islands. Secondly, it could be interesting to improve the efficiency of desalination plants and finally to introduce renewable energies to generate clean and emission-free energy [8,12].

## 4. Conclusions

Regarding the conclusions of this paper, the following is shown:

The main advantage of ecological footprint as an indicator is in the simple unit of measurement of this, the hectare, which allows us to make comparisons between different footprints in different places in the world.

In the Canaries it is necessary the desalination of large amounts of water to allow the development of the islands considering that almost all the electricity produced is from fossil fuels, it is about converting oil into water on a planet that demands to reduce emissions.

Water desalination in the Canary Islands requires more than 770,000 MWh, equivalent to more than 180,000 tons of fossil fuels each year (3600 barrels per day), at a cost of more than 100 million euros each year and the emission of more than 450,000 tons CO_2_ into the atmosphere each year.

It is obtained that the annual production of desalinated water in the Canary Islands is 216.41 hm^3^, for which it is necessary to use 1,058,240 MWh. Generating this volume of desalinated water generates a total of 694,445 tons of CO_2_.

The average consumption value of desalination plants in the Canary Islands is 4.89 kWh/m^3^. Granadilla de Abona desalination plant has a production capacity of 14,000 m^3^/d and the coefficient of emissions obtained in Spanish Electric Network for the day 13 October 2020 on the island of Tenerife is 0.525 t CO_2_/MWh.

Following the emission factor of 14.86 kg CO_2_/m^2^ and the area of the constructions of Granadilla de Abona desalination plant which is 2839 m^2^ built, 42.19 tons of CO_2_ are emitted for the construction of the different infrastructures of the desalination plant. Translated into global hectares (Gha) it would be 6060.5 Gha/year. Considering the size of the population related to Granadilla de Abona city, about 70,000 inhabitants, the ecological footprint is 0.065–0.087 Gha/inhabitant/year.

Energy consumption decreases when the feed temperature increases and when the feed salinity decreases too. Whenever possible it is interesting to introduce a higher percentage of renewable energies into the energy mix to reduce emissions and carbon footprint. With the introduction of photovoltaics, normally up to 30% of the energy needed by the plant can be obtained, and the remaining 70% is supplied by wind power when possible.

Canary Islands desalination carbon footprint is around 1203.84 tCO_2_/day, and the annual total is 439,402 tCO_2_. Therefore, the ecological footprint due to this is 219,701 ha/year. It means 0.1 Gha/inhabitant/year and the emissions per habitant and year are 0.2 tCO_2_/person/year, compared to 3.38 tCO_2_/person/year at the world level.

## Figures and Tables

**Table 1:** Average and equivalent CO_2_ absorption per hectare of the different surfaces of planet Earth. Surface area equivalence factors [8].

Category by Surface	ABS Average (tCO_2_/ha/year)	Surface (Millions ha)	%	Abs. Gha Equivalent (tCO_2_/ha/year)	Equivalence Factor (fi)
Forests	19.35	3858.10	7.56	1.46	9.66
Crops	8.09	1958.32	3.84	0.31	4.04
Meadows and pastures	2.44	3363.72	6.59	0.16	1.22
Oceans, seas, etc…	0.10	36,010.00	70.60	0.07	0.05
Deserts	0.00	3600.00	7.06	0.00	0.00
Others	0.00	2217.06	4.35	0.00	0.00
Total Surface		51,007.20		2.00	1.00

**Table 2:** Energy consumption of desalination in the Canary Islands.

Data of the Year 2015 Except Those of Tenerife (2012)	Annual Gridded Energy (GWh)	$\mathrm{Desalinated}\;\mathrm{Water}\;\mathrm{Production}\;(hm^{3})$	$\mathrm{Energy}\;\mathrm{Used}\;\mathrm{to}\;\mathrm{Desaturate}\;\mathrm{with}\;4.89\;\mathrm{kWh}/m^{3}\;(\mathrm{GWh})$	Percentage of Energy Used to Desalinate (%)
Gran Canaria	3376.68	78.15	382.15	11.31%
Tenerife	5571.04	26.64	130.27	2.34%
Lanzarote	817.23	24.4	119.32	14.6%
Fuerteventura	640.79	79.78	390.12	60.88%
La Gomera	69.23	6.07	29.68	42%
El Hierro	42.99	1.37	6.7	15.56%

**Table 3:** Emission coefficients per island (t CO_2_/MWh.) of the 13 October 2020.

Time (h)	Gran Canaria	Tenerife	Fuerteventura	Lanzarote	La Gomera	El Hierro
1:00	0.503	0.437	0.604	0.637	0.747	0.591
3:00	0.562	0.441	0.649	0.699	0.738	0.453
5:00	0.584	0.5	0.658	0.671	0.75	0.453
7:00	0.682	0.468	0.594	0.663	0.753	0.564
9:00	0.758	0.571	0.593	0.78	0.656	0.564
11:00	0.721	0.427	0.591	0.778	0.672	0.637
13:00	0.766	0.453	0.568	0.753	0.68	0.61
15:00	0.776	0.429	0.657	0.752	0.688	0.637
17:00	0.730	0.480	0.713	0.791	0.671	0.75
19:00	0.738	0.650	0.637	0.823	0.658	0.702
21:00	0.717	0.708	0.874	0.769	0.725	0.66
23:00	0.596	0.636	0.666	0.639	0.718	0.703
1:00	0.568	0.629	0.836	0.605	0.729	0.625
Average value	0.669	0.525	0.665	0.72	0.706	0.612

**Table 4:** Desalination emissions in the Canary Islands CO_2_ in 2015.

Data of the Year 2015 Except Those of Tenerife	$\mathrm{Desalinated}\;\mathrm{Water}\;\mathrm{Production}\;(hm^{3})$	$\mathrm{Energy}\;\mathrm{Used}\;\mathrm{to}\;\mathrm{Desaturate}\;\mathrm{with}\;4.89\;\mathrm{kWh}/m^{3}\;(\mathrm{kWh})$	Desalination Emissions CO_2_ by Island (t)
Gran Canaria	78.15	382.15 × $10^{6}$	255,658.4
Tenerife	26.64	130.27 × $10^{6}$	68,391.8
Lanzarote	24.40	119.32 × $10^{6}$	85,910.4
Fuerteventura	79.78	390.12 × $10^{6}$	259,429.8
La Gomera	6.07	29.68 × $10^{6}$	20,954.1
El Hierro	1.37	6.70 × $10^{6}$	4100.4

**Table 5:** Impact desalination plant.

Consumption of Natural Resources	Waste Production
Electricity Construction desalination plant	Reverse osmosis membranes Brine spills

**Table 6:** Global hectare values based on the ecological footprint calculated annually.

Type of Space or Activity	Gha
Desalination to 4.89 kWh/$m^{3}$	6060.5
Agriculture (mainlands)	13,393.71
Agriculture (marginal lands)	10,848.3
Forests	6060.5
Livestock	2969.65
Fishing (sea waters)	2181.78
Fishing (inland waters))	2181.78
Artificialized	13,393.71

## Data Availability

Not applicable.
